# Applying the greenhouse gas inventory calculation approach to predict the forest carbon sink

**DOI:** 10.1186/s13021-025-00307-2

**Published:** 2025-06-21

**Authors:** Fredric Mosley, Jari Niemi, Sampo Soimakallio

**Affiliations:** Finnish Environment Institute, Helsinki, Finland

**Keywords:** National inventory report, Greenhouse gas inventory, Forest, Sink, Carbon, Balance

## Abstract

**Background:**

Finland’s national Climate Act contains a target for carbon neutrality by 2035. Achieving this target not only depends on the effective implementation of emission reductions, but to a large part on the forest carbon sink. A recent publication of the Government’s analysis, assessment, and research activities highlights a potential disparity in forest land greenhouse gas (GHG) balance estimates by the ex-ante scenario model used in the National Energy and Climate Plan (NECP), and the ex-post GHG inventory methodology used for creating an official record of emissions and removals. Better methodological compatibility is needed to answer a key question: How large will the forest carbon sink be in different scenarios? This study is a first attempt to show the usefulness of applying the GHG inventory calculation approach to predict the forest carbon sink.

**Results:**

In this study, we introduce a tool that can be used to estimate the GHG balance for forest land, what we call a “synthetic inventory”, and validate it by comparing outputs against historical data reported in Finland’s GHG inventory. Second, we use it to predict GHG balances in year leading up to 2035 at various roundwood and forest residue harvest rates. The tool can replicate forest GHG balances for forest land with an average annual error of 1.0 Mt CO_2_, representing 4% of the average annual forest carbon sink. We estimate the forest GHG balance in 2035 to be around 3, -15, -32 Mt CO_2_eq at levels of total annual drain 92, 80, 70 Mm^3^ respectively.

**Conclusions:**

According to our calculations the forest land net GHG balance in 2035 is approximately 12 Mt CO_2_eq higher than what is presented in Finland’s NECP. Conceptual differences between how GHGI methodologies and scenario models estimate living biomass gains and losses contribute to this outcome, in addition to uncertainties associated with both approaches. The tool presented here shows agreement with the National Inventory Report 2023 approach for forest land, and it can be quickly updated to fit new data.

**Supplementary Information:**

The online version contains supplementary material available at 10.1186/s13021-025-00307-2.

## Background

Finland’s Climate Act contains a binding target for climate neutrality in 2035 [1]. In this framework, the Land Use, Land Use Change and Forestry (LULUCF) sector needs to provide a net sink to compensate for residual emissions. Assuming a linear reduction pathway in greenhouse gas (GHG) emissions (excl. LULUCF) between 2030 and 2040, residual emissions will be 21 Mt CO_2_eq in 2035 [1]. Forests, which cover around 21.7 million hectares in Finland, with 73% of the forest area on mineral soils, 8% on undrained organic soils and 20% on drained organic soils, have historically provided a strong net sink of around 30 Mt CO_2_eq per year in the period of 1990–2010 [2]. Over the past decade the forest sink has weakened, turning into a net source of GHG emissions [2]. The trend in net emissions can be explained by changes in tree growth, increased harvesting rates and increased CO_2_ emissions from forest drained organic soils [3]. If net GHG emissions in all sectors remain at this level, the 2035 target will be exceeded by over 50 Mt CO_2_eq [2].

Results from forest simulation models show that the forest carbon sink is largely affected by forest harvest [4]. For example, a systematic literature review showed that on average each additional unit of carbon harvested annually decreases the forest carbon sink by 1.6 (SD 0.9) units in comparison to a scenario with lower harvesting rate [5]. In the context of climate policy and achieving LULUCF targets, a key question is how large the forest carbon sink will be under any given harvesting rate, or on the other hand, how much wood can be harvested while still maintaining the required forest carbon sink.

The National Energy and Climate Plan (NECP) of Finland estimates the forest land GHG balance to be -8 and -27 Mt CO_2_eq at roundwood harvesting levels of 81.9 and 70 Mm^3^, respectively [6]. A challenge for planning and decision-making is that forest simulation models used in impact assessments, for example in the abovementioned NECP, often employ methods that cause their results to deviate from those reported in National Inventory Reports (NIR) [6, 7]. This may give the wrong signal about how the forest carbon sink as reported in NIR responds to specific harvesting rates. The challenge was obvious and identified when the European Union (EU) Member States proposed their forest reference levels in accordance with the EU LULUCF regulation between 2019 and 2020 [8]. The European Commission has recommended for Finland to ensure that the quantification of planned measures is consistent with greenhouse gas inventory estimates [9].

This study has two main objectives. The first is to create a tool that uses publicly available information to produce forest land GHG balance estimates comparable with Finland’s NIR [10]. For this aim we developed and tested a new ex-ante approach to estimating forest GHG balances at various forest harvesting rates. We validate the tool by estimating the historical GHG balances of forest land in Finland between 1990 and 2022 and comparing the results to those reported in NIR 2023 CRF-Table [11] and the latest available year from Statistics Finland [12]. The second objective is to compare the calculation results of the tool to the forward-looking estimates of the NECP [13]. We estimate the GHG balances in years leading up to 2035 at varying roundwood and residue harvesting levels.

## Methods

### Approach

IPCC Good Practice Guidelines assist countries in producing GHG inventories for various sectors, including the LULUCF sector [14, 15]. In accordance with these guidelines, Finland uses Tier 3 advanced methods and country-specific data to calculate GHG balances separately for living biomass, mineral soils and a Tier 2 method for organic soils on forest land [16]. With this as starting point, we aim to create a tool that uses publicly available data, is easy to update and produces forest GHG balance estimates that are comparable with estimates made using official methods of NIR 2023 [16].

To estimate the living biomass CO_2_ balance, we combine living biomass CO_2_ gains and losses data from NIR [11, 12] with statistics describing the difference between gross annual increment data and total drain (total roundwood harvest, logging residues, natural mortality) [17] in a regression model. For mineral and organic soil emission calculations we use the soil model Yasso07 [18] and the calculation approach by Alm et al. [19]. To predict GHG balances in years leading up to 2035, we adopt values for growing stock, net annual increment, harvest, and total drain used in the NECP [6] (Table 1). A detailed description of the tool and its data needs is provided in the next section, and all data and calculation steps as well as a flow chart are included in the online supplementary materials, except for the simulations which were done with the soil model Yasso07.

**Table 1:** Scenario data used for calculating living biomass CO_2_ balances

	2023–2028	2029–2035	2023–2028	2029–2035	2023–2028	2029–2035
Scenario	WEM-P	WEM-P	WEM-L	WEM-L	Drain 70	Drain 70
Net annual increment, Mm^3^	103.7	103.2	104.8	106.6	107*	109.6*
Total drain, Mm^3^	88.9	92.3	82.6	80.4	70	70
Roundwood harvest, Mm^3^	77.3	81.9	71.1	70	58.6*	58.6*

*Net annual increment was estimated by extrapolating from WEM-P and WEM-L scenarios. Roundwood harvest was calculated from the drain by applying the average ratio of roundwood harvest to total drain over the period 1990–2022

### Tool description

#### Living biomass

NIR CO_2_ balance estimates for living biomass are based on the difference between gains and losses [16]. Where the gain-component of living biomass is the gross increment, i.e. increase in dry matter of living trees before accounting for harvest and other losses. This is based on measurements carried out in National Forest Inventories (NFI) and estimations of biomass accumulation in tree compartments using Biomass Conversion and Emission Factors (BCEFs). Losses, on the other hand, are estimated by combining statistical surveys for total roundwood harvest volume and estimates for losses due to natural causes e.g., disturbances and mortality, and converting the volumes to biomass using BCEFs [16]. The statistics on total roundwood harvest also include biomass resulting from deforestation, but the deforested area is not included in the total forest land losses data. We deducted the share of roundwood from deforestation from the total roundwood harvest. The amount was estimated by comparing the share of losses that occur when forest is converted to other land uses reported in CRF-Table [11] to total drain [17].

**Fig. 1:**
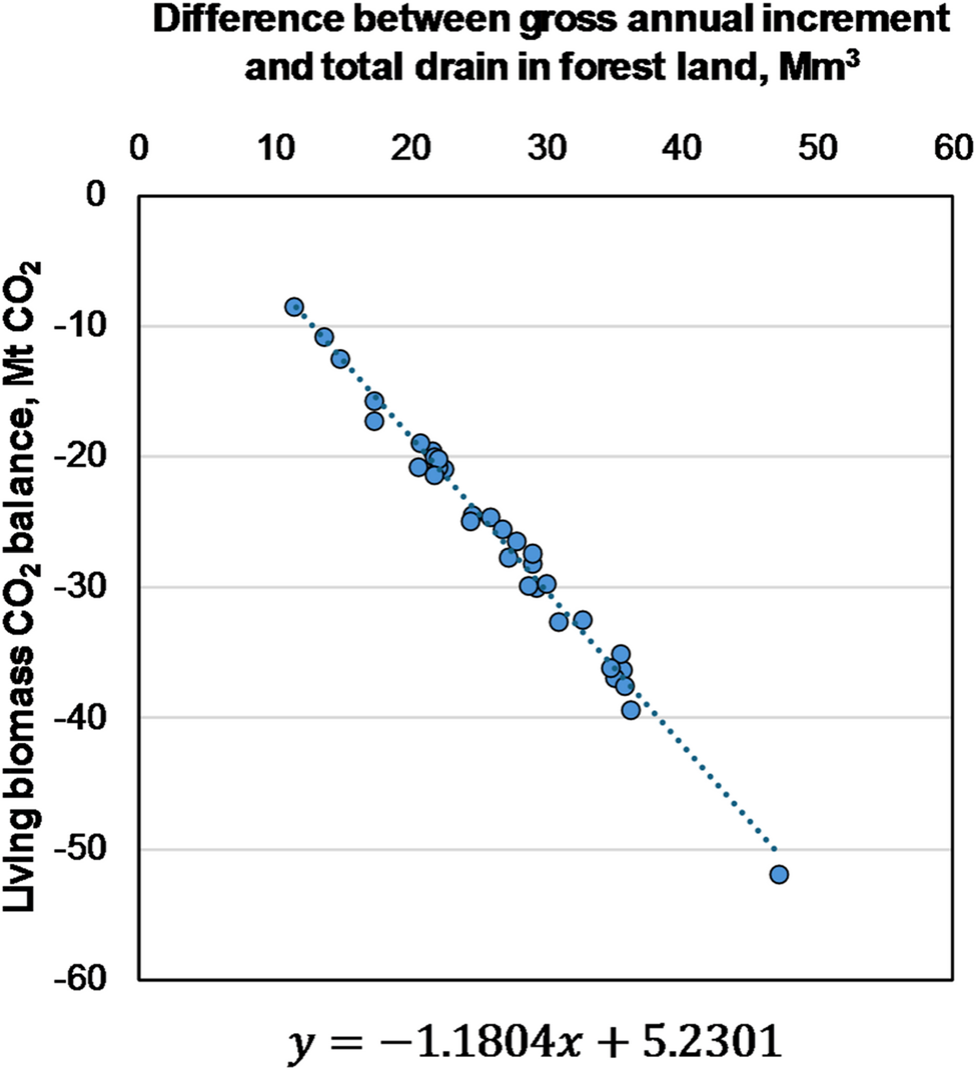
Empirical regression model that relates historical (1990–2022) CO_2_ balances reported in NIR CRF-Tables [11, 12], and the difference between gross annual increment and total drain in forest land based on National statistics [17] (R^*2*^ = 0.983; ρ < 0.00001)

Resultingly, the annual CO_2_ balance of living trees, in this case the dependent variable, is strongly, positively correlated with the difference of annual increment (Mm^3^) and total drain (Mm^3^), the independent variable. Some temporal variation is expected because of differences in data. For example, because NFI-based BCEFs change over time. To estimate the living biomass CO_2_ balance for a given difference between gross annual increment and total drain in forest land, we use this linear regression model (Fig. 1).

#### Mineral soil

In the NIR 2023 approach for mineral soils, annual CO_2_ emissions and removals are calculated with the stock-difference method [16]. To replicate the calculation approach, we needed data for litter input from living trees, harvesting residues, natural drain, and weather data for South and North Finland, going back to years 1971 and 1975, respectively.

First, litter input from living biomass is calculated for South Finland and North Finland separately, and by source: living trees, ground vegetation, harvesting residues and natural losses. Litter is also divided into groups based on diameter and chemical quality: 0 cm non-woody litter (foliage, fine roots), 2 cm fine woody litter (branches, coarse roots) and 10 cm coarse litter (stem and bark). Decomposition is then estimated using the soil model Yasso07 [18], initialized from year 1971 for South Finland and 1975 for North Finland. Yasso07 is freely available for download [20] and the NIR approach can be replicated with some data preparation described next.

To obtain the annual litter input from living trees, we converted values for total growing stock volume on forest land and poorly productive forest land to mean growing stock, and further to biomass using tree species and biomass component specific BCEFs [21]. Annual litter input from living trees was obtained by multiplying biomass components (foliage, branches, bark from stems, coarse roots, fine roots, bark from stumps) with their respective litter production rate coefficients. Publicly available species-specific information for growing stock volume based on soil types is currently limited to the years 2009–2022 [22]. Therefore, we derived growing stock volumes for 2004–2008 from literature [23], and earlier years from statistical yearbooks [24]. The distribution of growing stocks across mineral soil and organic soil before 2003 was estimated by combining sources [23, 25].

Estimates for natural drain were taken from NIRs [26, 27] and statistical yearbooks [24], after which they were distributed across regions and soil type based on growing stock volume and divided into components (foliage, stem, branches, stump, coarse roots, fine roots). To form forward looking estimates, the growing stock is interpolated from the first year of NFI 12/13 (2018) to 2029, which is the next data point available from NECP scenarios [6]. Litter input from harvesting residues, i.e., biomass that is left on site to decompose, was derived by distributing total roundwood harvest [17] across North Finland, South Finland and soil types based on Statistical Yearbooks [24], converting the harvest volumes to biomass (foliage, branches, waste wood, stump) using BCEFs [28] and subtracting biomass in residues harvested for energy [24]. Waste wood was considered to consist of stemwood and was treated accordingly in Yasso07. The share of harvests on mineral soils was approximated through the proportions of harvested area between mineral soils and organic soils in NFI 10 [23], and assumptions for the geographic distribution of residue harvest based on the MELA model used in NECP [29].

Litter production rates and the Yasso07 parameter set were obtained from NIR 2023 [16]. Litter input from ground vegetation in North Finland and South Finland was calculated by multiplying ground vegetation production rate coefficients [16] with the area of mineral soil, which was obtained from statistics for years 2009–2022 [30]. For earlier years the mineral soil area for whole Finland were obtained from NIR 2023 CRF-Table [11], and divided into South Finland and North Finland based on the ratio in year 2009 [30]. The chemical composition (AWEN) of litter groups (non-woody litter, fine woody litter, coarse litter) and weather data (temperature and precipitation) used for calculating 30-year moving averages (e.g., 1988 to 2017 for 2017) was obtained from Alm et al. [19]. For North Finland and South Finland, the forward-looking estimates for temperatures were calculated by using AWEN from the latest available year and continuing the 30-year moving average without adding new data after 2022. Finally, yearly carbon stock changes were calculated with Yasso07 using the annual litter inputs, weather data and litter quality, before converting the results to CO_2_ and then calculating five-year moving averages, as is done in the NIR 2023 [16].

#### Organic soil

The NIR 2023 approach for estimating carbon stock changes on drained organic soils is based on the difference between litter input and decomposition, and it is documented in Alm et al. [19]. Similarly, as for mineral soils, the results can be replicated with a few modifications and data preparation steps.

We estimated litter input from living trees and harvesting residues as we did for mineral soils, with slight modifications. Fine roots biomass was excluded and calculated separately. We also reduced the share of organic soil biomass that is on drained organic soils by 10% in NFI 12, 20% in NFI12/13 and 25% thereafter. This is because we observed litter input was being increasingly overestimated after 2009. A likely explanation is that more forest stands on drained organic soils are being harvested as they are reaching final felling age [19], and the share of growing stock is decreasing. However, this data is not available. The harvested area was adjusted to fit harvesting residues litter input from Alm et al. [19]. Yasso07 was initialized over a period of 50 years using the average natural drain and harvesting litter input from 1975 to 1977, after which annual litter inputs were used [16].

The remaining calculation steps followed the NIR 2023 approach [16]. Arboreal fine root litter input was determined for each drained peatland forest type (FTYPE) separately using regression models that use mean basal area and mean dwarf shrub cover as predictors [19]. Litter input from ground vegetation for South and North Finland and different FTYPEs was calculated using regression models that use mean basal area as a predictor [19]. Decomposition of peat and litter (excl. harvesting residues and natural drain) was calculated for each FTYPE separately using regression models that use basal area (m^2^/ha) and mean May-October air temperature as predictors. This contains the decomposition of the abovementioned litter input (living trees, arboreal fine roots, ground vegetation). The basal area for the years 2024–2035 needed to be approximated, and we did so by relating the living trees litter input to basal area in year 2021 and assuming a stable relationship. Areas and data for chemical quality and weather data for the historical period, incl. May-October mean air temperature for forest types was obtained from Alm et al. [19]. Outside the historical period 1990–2022, May-October mean air temperature for FTYPEs were kept constant at the level of the nearest available year. The 30-year moving averages of temperature and precipitation needed for Yasso07 were calculated in the same way as for mineral soils. Finally, annual carbon stock changes were determined by calculating the difference between litter input and decomposition, before converting to CO_2_. Estimates for CH_4_ and N_2_O emissions from drained peatlands from NIR [16] and NECP [6] were then added to the balance.

#### Other greenhouse gases

Besides CO_2_, NIRs include the emissions of non-CO_2_ greenhouse gases [10]. CH_4_ and N_2_O emissions from drained peatlands in Finland have declined since 1990 and stabilized around the level of 2.43 Mt CO_2_eq per year [11]. Emissions from N fertilization, biomass burning, and N mineralization are stable and have a marginal effect on total GHG emissions [11]. These estimates are used for the historical period in the tool. For scenario calculations we derive non-CO_2_ emissions from drained peatlands from NECP [6], and other emissions were assumed to remain at the average levels of 2018–2022 [11].

### Scenarios

Scenario data needed to make forward-looking estimates was taken from the NECP [6]. We selected two ‘With Existing Measures’ scenarios: WEM-P (basic) and WEM-L (low). The aim of WEM-P and WEM-L scenario was to provide a GHG emission pathway with moderate (WEM-P) and lower (WEM-L) economic development to inform climate policy. To illustrate the versatility of the tool, a third scenario with smaller harvest rate, so-called drain 70 Mm^3^, was added (Table 1). The annual increment of Drain 70 Mm^3^ scenario was derived assuming linear relationship between annual increment and total drain based on WEM-P and WEM-L scenarios. The annual harvest rate of Drain 70 Mm^3^ scenario was derived by using the average ratio (1.19) between total drain and harvest rate in 1990–2022.

### Assessing the accuracy of the tool

We evaluated the accuracy of GHG balances for living biomass, mineral soil and organic soil produced by our tool over the historical period 1990–2022 and predicted period 2023–2035. Comparing against historical GHG balances [11, 12] enabled us to assess if the tool has the accuracy needed for the intended application. Predicted values were compared to estimates used for the National Energy and Climate Plan [6]. The focus of the evaluation was on identifying possible errors caused by differences in data used by the tool and NIR 2023 [16]. The structural uncertainty of the inventory methods to describe physical and biological processes were not analyzed here.

## Results

### Evaluation of the calculation approach

**Fig. 2:**
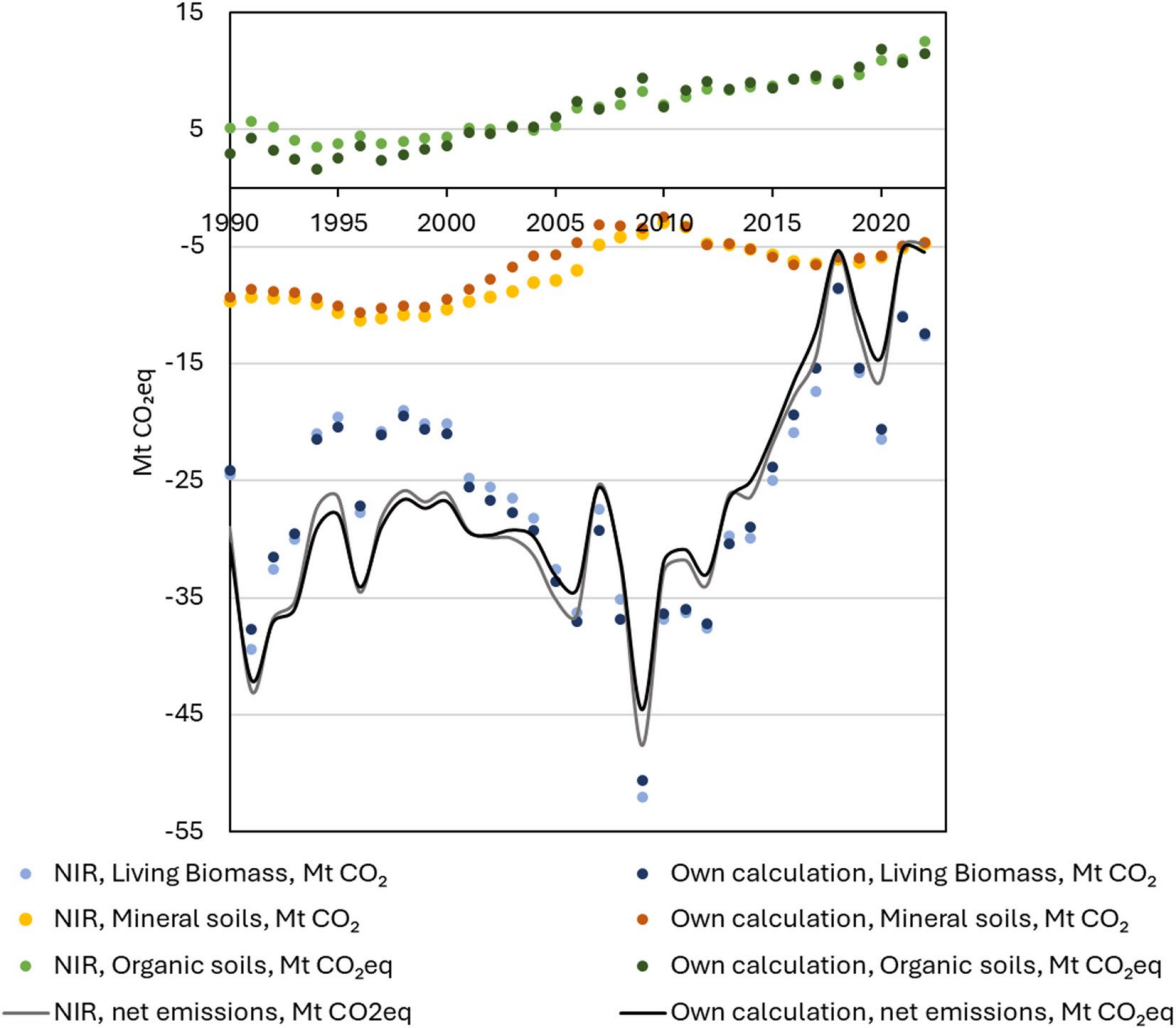
NIR living biomass, mineral soil, and organic soil GHG balances [11, 12] compared to estimates produced using the tool for years 1990–2022

The annual GHG balances for living biomass, mineral soils and organic soils calculated using the tool over the historical period 1990–2022 were compared against the corresponding GHGI values taken from CRF-Tables [11, 12]. As shown in Fig. 2, the tool shows agreement with the NIR [11, 12] and traces the total forest land net emissions with annual errors ranging from 0.12 to 3.02 MtCO_2_eq. The average annual difference in carbon balance when comparing the results from the tool and NIR over the historical period was 0.84, 0.74, 0.79 and 1.00 Mt CO_2_eq for living biomass, mineral soil, organic soil, and total forest land, respectively. This represents 3%, 10%, 20% and 4% of the average absolute annual GHG balance in each compartment. The estimates for living biomass are consistent over the whole period, while errors in soil estimates fluctuate more in years before 2009 when data availability is lower.

Some simplifications were made to the calculation steps due to data gaps, and next we discuss possible sources of error between our results and NIR. In estimating the living biomass sink, we avoid the need for granular NFI data by calculating the sum of gains and losses at national level by using the historical relationship between biomass and CO_2_. We find that the relationship between the sink and difference between annual increment and total drain is well approximated (R^2^ = 0.983) and statistically significant (ρ < 0.00001). It holds true for the observed range where the difference is between 10 and 50 Mm^3^. One possible explanation for errors here is the lack of consideration for dynamic changes in the relation between annual increment and drain, and BCEFs used for converting to CO_2_.

The methods for calculating mineral soil and organic soil CO_2_ balances are similar to the NIR approach, yet there are some differences in calculation steps and input data. Errors in soil sink estimates over the historical period arise when approximated litter production does not perfectly match the official values. For example, by comparing our living biomass estimates against statistics publications [31], we find that the stock in our calculations may be underestimated for mineral soils.

The tool derives growing stock volume by combining soil type area information and growing stock data for forest land and poorly productive forest land. Therefore, the growing stock estimate for organic soils will be different than what is expected to be found on drained peatlands [24]. We found that the litter input from living trees on drained organic soils was overestimated in years after 2009 and it was corrected by assuming that the share of growing stock outside drained peatlands increases. To our understanding the diameter classes, chemical composition (AWEN), temperature and precipitation data in the tool are similar to those used in NIR 2023 [16], but differences exist as data has been aggregated and analysis carried out at lower level of granularity.

A simplification that affects all components is the treatment of land use categories. In the tool we only consider total forest land, while NIR 2023 [16] divides forest land into two categories: (1) forest land remaining forest land, (2) land converted to forest land. The motivation for our choice is that area fluctuations in total forest land and its effects on the total CO_2_ balances are relatively small (land converted to forest land contributed about 2% of the living biomass sink in 2022 [2]) and expanding the approach would increase the need for data that is not publicly available. Exclusion of these underlying drivers seems to only have minor effects on the tools ability to produce NIR comparable GHG balances throughout the historical period.

### Predictions made using the tool

To test the tools applicability for making forward-looking predictions we calculated GHG balances in three scenarios: (1) WEM-P and (2) WEM-L from NECP [6], and (3) a scenario where total annual drain remains at a level of 70 Mm^3^. Additionally, we tested two variations in the rate of collecting forest harvesting residues in relation to the WEM-P scenario.

**Fig. 3:**
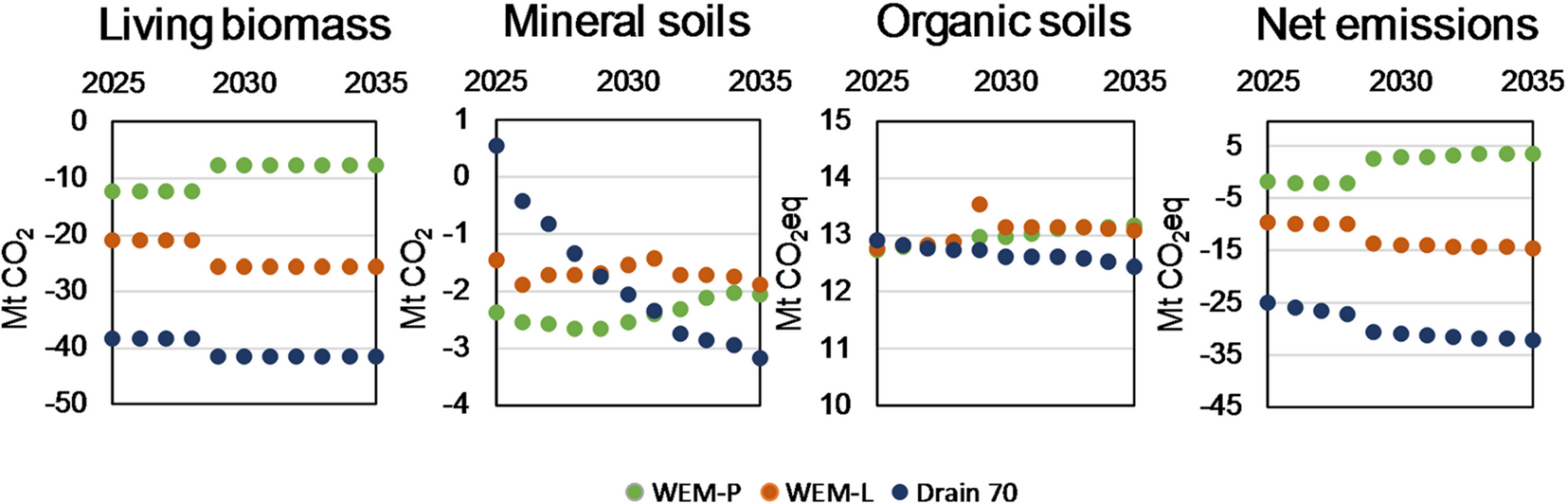
GHG balances for living biomass, mineral soils, organic soils and total forest land in three scenarios calculated using the tool

Emissions from mineral soils remained at a stable level in all scenarios except for (3) where lowering of the harvest rate first caused a peak in emissions from the soil when the emission output from decaying organic matter from recent years was not compensated by the new harvesting residue input. After the peak, the emissions from soil stabilized at a lower level. The dependence of the emissions from organic soils on temperature resulted in an increasing trend in emissions from organic soil, while lowering the harvest rate resulted in more carbon in the forest, seemingly at least in the considered time frame compensating for emissions from other components (See Fig. 3).

Using the WEM-P scenario data for annual increment, total drain, total roundwood harvest and growing stock volume, the tool predicts that Finland’s forest land will be a net source of 3 Mt CO_2_ in 2035. Lowering total drain from more than 90 Mm^3^ (harvest rate more than 80 Mm^3^) to approximately to 80 Mm^3^ (harvest rate to 70 Mm^3^) strengthened the sink to around -15 Mt CO_2_eq, while a level of 70 Mm^3^ total drain (60 Mm^3^ harvest rate) resulted in a sink around -32 Mt CO_2_eq in 2035.

The net emissions in 2035 calculated for WEM-P scenario using the tool results in considerably higher values than those reported in the NECP [6] report (Table 2). This is mainly due to a gap in living biomass sink estimates, which is around 10 Mt CO2eq in the WEM-P scenario. The difference in mineral soil estimate is 2.1 Mt CO_2_, and 0.4 Mt CO_2_eq in organic soils.

**Table 2:** Comparison of 2035 GHG balance for WEM-P by NECP [6] and the tool

GHG balance 2035	NECP	Own calculation
Living biomass, Mt CO_2_	-17.7	-7.6
Mineral soil, Mt CO_2_	-4.2	-2.1
Organic soil, Mt CO_2_eq	13.5	13.1
Net emissions Mt CO_2_eq	-8.3	3.5

For soils, NECP [6] uses the same stock difference approach with Yasso07, and the Alm et al. [19] calculation method for organic soils. Other gaps between estimates could be explained by the different data, simplifications, and modified calculation steps in the tool. This includes BCEFs which estimate a lower input of biomass than what is calculated by NECP. In organic soils the final difference is small, but it should be considered that the tool result is uncertain because of lack of accurate data about the share of growing stock that is located on drained organic soils.

**Fig. 4:**
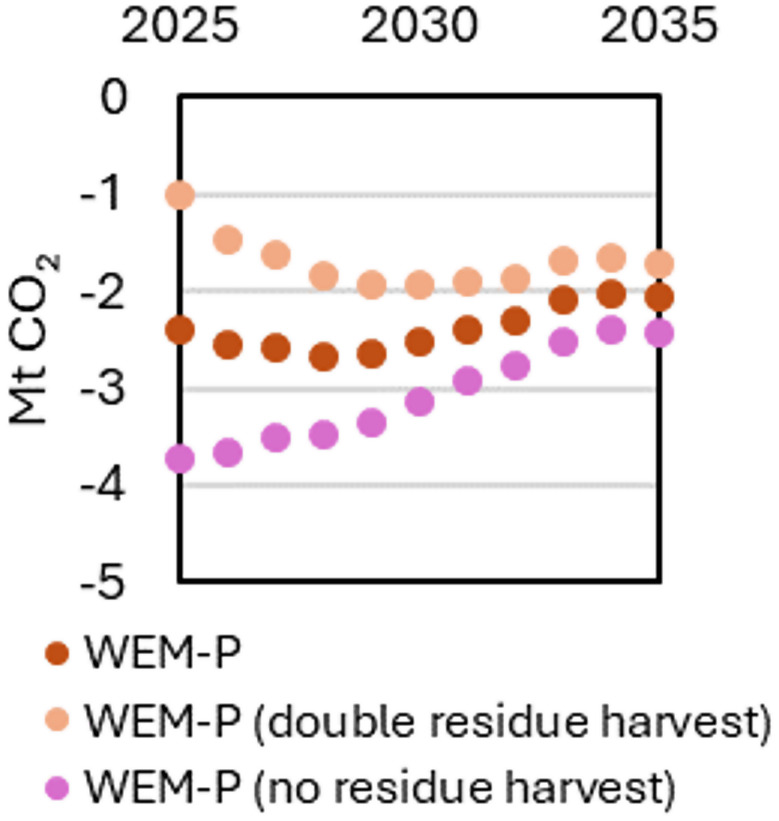
Effect of residue harvest on the mineral soil GHG balance illustrated by varying the collection of harvesting residues (foliage and branches) in the WEM-P scenario

We also tested varying the rate of collecting harvesting residues (foliage and branches) in the WEM-P scenario. The collection of waste wood was not changed as it is incorporated in the total roundwood harvest of NECP scenarios, and stump harvest was left at the 2022 level as stump harvesting is limited by the aims to conserve biodiversity. Based on data from MELA [29], it was assumed that all residue harvest only takes place on mineral soils, primarily in South Finland. Consequently, the effect can be observed in the Yasso07 model output for mineral soils (Fig. 4). Increasing extraction of harvest residues results in less carbon left in the forest, weakening the sink throughout the considered timeframe. However, due to relatively rapid decay of foliage and branches, the effect of increased collection of harvest residues abates quite strongly by 2035.

## Discussion

The present study suggests that synthetic inventories can be useful for predicting forest carbon balances, if they estimate carbon stock changes in a similar way they would be calculated and reported by countries in National Inventory Reports. We are not aware of any previous attempts apply an inventory calculation approach to scenarios for estimating forest land CO_2_ balances that are comparable with National Inventory Reports to the UNFCCC.

### Usefulness of the calculation approach

By highlighting the conceptual differences between calculation approaches, our tool contributes to improving comparability of forest sink estimates produced by different models and those reported in NIR. The approach complements conventional forest and ecosystem models as it does not include a growth model, but it relies on this data as input. It also shows the challenges with replicating the NIR calculations. Increased transparency and accessibility with regards to data (area information, distribution of growing stock, and BCEFs), as well as assumptions with regards to where harvesting takes place would be welcomed next step, which would further improve the accuracy of the tool.

The agile characteristic of this calculation approach can be particularly useful for dealing with uncertainty and long cycles in which NFI data is published. The tool is easy to update and improve. This is important considering that all future estimates are uncertain and depend on outdated information, other models and methodologies that may change, as well as errors. It is likely that corrections can be made to account for quick changes in assumptions and data, for example to include storms, drought, harvests, and other disturbances that occur in one year, but which effects will be spread out over many years of the five-year NFI period. Essentially it is the use of gain-loss method for living biomass which allows countries to pick up on quick changes in gains and losses, and it is therefore also a highly important methodological choice for NIRs. The alternative stock-difference (also known as stock change) method uses NFI data to calculate the size of the whole CO_2_ stock and compare size in two points in time. While the estimates derived using the stock-difference method are of low uncertainty, they are not able to provide information on the amount and type of harvesting [32]. The different timing and quality of data updates in stock-difference and gain-loss methods raises concerns about the monitoring of contributions to the shared EU GHG budget and sink target outlined in the LULUCF regulation. In Finland, annual increases in losses have been picked up by the gain-loss method and are reflected as a reduction in sink in the inventory, while for example in Sweden which applies the stock-difference method, upward corrections have been made to the living biomass sink later, when losses that have already occurred in previous years are detected [33].

Most European countries are in a similar situation as Finland. Forests are in an intermediate growing phase where annual tree increment is the highest [34], and the rate of losses has a strong effect on the size of the annual LULUCF carbon sink. The approach developed here could possibly be relevant to apply for other countries which use the gain-loss methods for calculating living biomass CO_2_ balances. We find that information about increment and drain is available from National statistics and other sources [35]. BCEFs and other data needed for calculating soil carbon stock changes are also available for many countries [10, 36].

### Putting the results into context

Finland’s living biomass and mineral soil carbon pools have been net sinks, while organic soil has been a net source of emissions since the beginning of GHG reporting in 1990 [2]. The WEM-P projection in the National Energy and Climate Plan [6] anticipates that the living biomass balance will remain stable into the future, while emissions from organic soils will increase and the mineral soil sink will become stronger (Table 2). When comparing to estimates produced using our tool, NECP seems to overestimate the living biomass sink. If the sink remains proportional to the difference between growth and total drain, our tool estimates the living biomass sink closer to -7.6 Mt CO_2_eq, instead of -17.7 Mt CO_2_ presented in NECP. At this lower level of living biomass sink, and if the emissions from mineral soil continue to increase, then forest land will be a net source (3.5 Mt CO_2_eq) of emissions in 2035. One explanation for the discrepancy in living biomass is the difference in the ratio between biomass and roundwood volume in statistics and that of the MELA model used in NECP. On the side of gains, the ratio between NIR biomass gains (Mt CO_2_) from CRF-Tables [11, 12] with volume increment (Mm^3^) from statistics [17] was 1.21–1.25 (average 1.23) for the (1990–2022), while the corresponding ratio for the MELA model was 1.32 for the periods 2019–2028 and 2029–2038 [29]. On the side of losses, the ratio between biomass losses (Mt CO_2_) [11, 12] and our estimated volume drain in forest land (Mm^3^) was 1.30–1.34 (average 1.32) for statistics (1990–2022) and 1.29 for MELA for the periods 2019–2028 and 2029–2038 [29]. This indicates that MELA generates more biomass in gains per volume tree growth and less biomass in losses per total volume drain than statistics, resulting in higher living biomass sink compared to statistics.

Finland’s LULUCF net sink target for 2035 is -21 Mt CO_2_eq [1]. Considering that net emissions from other land use categories (i.e., croplands, grasslands, wetlands, settlements, and harvested wood products) are expected to be around 5–10 Mt CO_2_eq [6], it means that forest land needs to provide a net sink between of at least -26 to -31 Mt CO_2_eq in 2035. Out of the three scenarios included in this study, only the Drain 70 scenario resulted in a net sink (-32 Mt CO_2_eq) exceeding this range. The other two scenarios (WEM-P and WEM-L) did not reach this required range with net balances of 3 and -15 Mt CO_2_eq in 2035. Considering the difference in forest land net sink and total drain between WEM-L and Drain 70 scenarios, we estimate that a forest land sink between -26 and -31 Mt CO_2_eq would be achieved with roughly 71–74 Mm^3^ total drain. Based on statistics this would correspond to approximately 60–62 Mm^3^ annual roundwood harvest rate.

## Conclusions

In the next years better methodological compatibility between models and GHG inventories is needed to answer a key question: What forest harvest level is compatible with climate targets? This study is a first attempt to show the usefulness of applying the GHG inventory calculation approach to predict the forest carbon sink.

We provide a tool for estimating annual GHG balances for forest land in Finland. The tool relies on publicly available data and can be easily updated to fit new information and accommodate changes in underlying methods. The calculation approach was able to trace the NIR 2023 [16] with a relatively minor error. We argue that our tool accurately estimates forest land sink for the near future with a given total drain and harvest rate. Technically, the tool can be applied to longer time horizons, but because of uncertainty in key factors including annual increment, species composition, age structure and BCEFs, the accuracy over decadal time horizons is also uncertain.

Our calculations show that the 2035 GHG balance of forest land is approximately 12 Mt CO_2_-eq higher (the sink is weaker) than what is currently estimated in Finland’s NECP scenarios. This gap corresponds to around 50% of the expected LULUCF contribution to the climate neutrality target of Finland. Conceptual differences between how GHGI methodologies and scenario models estimate living biomass gains contribute to this outcome. There are underlying uncertainties in all the parameters and methods of both NECP and NIR approaches and investigating them would require further studies.

## Electronic supplementary material

Below is the link to the electronic supplementary material.

Supplementary Material 1

## Data Availability

All data and calculations (excl. Yasso07 simulations) can be found in the online supplementary materials.
